# Supplementary material for: Are carbon emissions trading and green financial instruments synergistic? -Comprehensive quantitative research based on content analysis
Source: PLoS One. 2024 Mar 7;19(3):e0298601. doi: 10.1371/journal.pone.0298601 (PMC10919601; doi:10.1371/journal.pone.0298601)
Supplement: S1 Data — (ZIP) [file pone.0298601.s001.zip › Data/地级市Z_F工作报告.zip!/╕ú╜¿/─■╡┬╩╨/└┤╫╘╣1⁄2╓┌║┼í╛┬φ┐╦╩2╛▌═°í┐_2005.docx]

各位代表：

现在，我代表宁德市人民政府向大会作政府工作报告，请予审议，并请市政协委员和其它列席人员提出意见。

一、以科学发展观为指导，经济社会加快发展的一年

2004年，是我市树立和落实科学发展观，建设海峡西岸经济区东北翼增长极港口中心城市，国民经济和社会发展取得重要成效的一年。在省委、省政府和市委的坚强领导下，全市各级政府认真贯彻党的十六大和十六届三中、四中全会精神，积极贯彻国家宏观调控政策，努力消除干旱、台风等自然灾害影响，克服用地、资金和电力紧张等诸多困难，统筹协调，致力发展，经过全市人民共同努力，实现了市一届人大六次会议确定的国民经济和社会发展的各项目标。全年实现生产总值309.9亿元，增长11.2%；人均GDP突破1万元。全社会固定资产投资突破百亿元大关，达101.9亿元，增长37.1%。外贸出口2.6亿美元，增长25.7%。实际利用外商直接投资突破1亿美元，增长31.8%。财政收入15.8亿元（不含基金收入），增长12.1%，其中地方财政收入10.2亿元，同口径增长12.6%。社会消费品零售总额增长13.2%，居民消费价格总水平上涨4.2%。城镇居民人均可支配收入增长12.1%。农民人均纯收入增长8.4%。城镇登记失业率控制在4.3%以内，人口自然增长率5.6‰。其中生产总值、全社会固定资产投资、社会消费品零售总额、实际利用外商直接投资、城镇居民人均可支配收入、农民人均纯收入增幅都创1998年以来最好水平。全市正在形成风正气顺、人和业兴的创业趋势。一年来的主要工作和成效是：

1、立足长远发展，不断创新发展思路和举措。积极呼应全省建设海峡西岸经济区的战略部署，提出了建设海峡西岸经济区东北翼增长极港口中心城市的战略定位和目标，进一步明确了我市今后一个时期的发展目标、布局和举措。超前谋划发展规划，编制了中心城市总体规划和控制性详规、港口开发规划、临海产业发展和布局规划、临海化工产业规划、临海钢铁产业规划、旅游规划和交通路网规划等一批重要规划。超前谋划了一批战略性交通重大项目，宁上高速公路列入交通部规划，宁德至浙江衢州铁路列入部、省规划考虑，古田接京福高速连接线、福安经寿宁接泰顺高速也在规划中，还有一批连接省内外的交通干线及大型临海项目的建设和前期工作也取得重大进展，为延伸"两翼"、对接"两洲"、拓展腹地创造条件。

2、推动产业发展，经济增长的速度、质量和效益同步提高。农业农村经济较快增长。农林牧渔业总产值131.7亿元，增长5.1%。粮播面积扩大7万多亩，粮食生产扭转连续四年减产局面，总产量增长6%。茶叶、食用菌、水产、畜牧、果蔬等主导产业不断发展壮大。农业产业化步伐加快，新增绿标产品5个，无公害农产品8个，有15家企业被确认为省级农业产业化重点龙头企业，南阳实业公司成为我市首家国家级农业产业化龙头企业。工业经济不断提升。完成工业总产值261.7亿元，增长16.4%，其中规模以上工业产值128.4亿元，增长29.2%，占全市工业产值的比重从上年的40%提高到49.1%。新增规模以上工业企业81家。实施工业技改项目134个，完成投资14.8亿元，增长51.3%，增幅列全省第一。商贸流通业加快发展。全市交易额上亿元的商品市场达7家，社会消费品零售总额达97.7亿元。旅游业发展形势喜人。全年接待游客306万人次，旅游总收入20.7亿元，分别增长20%和16%。白水洋成为全省旅游八大品牌之一，福安白云山、寿宁杨梅洲新评为省级风景名胜区。金融运行态势良好。全市金融机构本外币各项存款余额194.3亿元，贷款余额172.2亿元，分别增长15.2%和20.2%。房地产、运输业、邮电通信等稳步发展。

3、突出项目带动，重点建设取得新突破。温福铁路、霞浦机场正式开工。城澳一类口岸通过省级验收。去年确定的39个市级重点建设项目中，已有宁德城澳万吨码头、三沙供水工程等7个项目竣工，穆阳溪梯级二级周宁水电站等5个项目完成主体工程，福安白马10万吨级船坞等4个预备重点项目实现开工建设。其他项目也基本完成年度计划。电网改造建设加快推进，完成了福州到宁德及大部分县（市）的双回路输变电线路建设，解决了我市长期以来电网脆弱、承受力低下的矛盾。同时，去年确定的83个中心城市建设项目也取得较大进展，有24个项目竣工或完成年度计划，27个项目实现动工，还有32个项目也已完成勘探、设计等前期工作，今年开工建设。

4、深化改革开放，经济发展动力和活力进一步增强。工业企业产权改造、资产重组深入推进，全市80%以上的内贸企业进行了改革。电力供电体制改革全面完成，成为全省唯一完成县（市）供电公司股份制改革任务的设区市。开发区清理整顿工作基本达到预期目标。事业单位聘用制改革试点也取得实质性进展。中心城市管理体制改革逐步到位，运行机制更加完善。农村改革继续深化，下派610名干部驻村任职，推动农村工作开展。有1177个村基本完成林改任务，完成人工造林更新10.5万亩。全面落实农村税费改革和种粮补贴等措施，全市农民直接受益3.8亿元，是农民得到实惠最多的一年。招商引资力度加大，先后在海内外组织开展了多场招商推介活动，成功举办了第五届宁德招商节及茶叶、食用菌、旅游文化节等一系列招商活动，宁德在国内外的知名度进一步提高。共签约项目208个，其中外资项目51个，合同外资金额4.98亿美元，比上年增长109%，全年新增外商投资企业48个，投资1.87亿美元；内资项目157个，合同内资金额81.95亿人民币。对台经贸往来有新进展，城澳4号码头获准以个案形式直运石料至马祖并成功直航。

5、坚持以人为本，城乡人民生活水平稳步提高。城镇居民人均可支配收入8985元，增加971元；农民人均纯收入3517元，增加271元。就业再就业工作继续推进，全年新增就业2.6万人，有7200多名下岗失业人员实现再就业，新增农村劳动力转移6.7万人。社会保障覆盖面进一步扩大。发放机关、企事业单位离退休人员和农村参保人员基本养老金2.46亿元，失业人员失业金0.27亿元，为全市2.58万名统筹企业退休人员增发基本养老金，月人均增发33元，有14.5万名职工享受医保待遇，并落实城镇居民低保对象2.24万人，农村家庭年收入低于1000元的低保对象10.97万人纳入低保范围，比省核定数增加1.37万人。农村生产生活条件继续改善，完成乡村供水工程236个，硬化农村道路650公里。防抗禽流感、森林防火、防汛抗旱等工作取得实效。面对"艾利"强台风，责任到位、部署周密、措施有力，保证了群众生命财产安全，最大限度降低了灾害损失。重视灾后重建工作，受灾倒房户春节前全部搬进新居。扶贫开发取得新成效，落实省、市扶贫开发重点村扶贫项目276个，下拨捆绑资金1100万元。造福工程和滑坡险情村搬迁7500人，超额完成省下达任务的50%。华侨农场职工安居工程已竣工266套，华庭小区市直单位住房已竣工206套。年初确定的16件为民办实事项目基本完成。做好清理建设领域拖欠工程款和农民工工资，完成了省里确定的清欠目标。

6、注重统筹协调，各项社会事业加快发展。科技工作进一步加强，启动"科技兴业"示范工程，颁布实施《宁德市科学技术奖励规定》，设立科学技术"杰出人才重大贡献奖"，全年实施部、省科研项目33项。"两基"成果不断巩固，高中阶段教育发展加快，高中在校生达9.6万人，比增17.2%。人才强市战略深入实施，制定出台了《关于进一步加强人才工作的若干意见》。公共卫生事业稳步发展。启动33个乡镇卫生院危房改造，着手解决341个农村卫生所"空白点"村建设补助和村医补贴，市县两级疾控中心和传染病区都在加快建设。文化事业加快发展，畲族民间文化和文物抢救保护工作力度加大，一批文艺作品获得国家级奖项。群众体育、竞技体育继续发展。计生工作连续十年实现省下达的人口控制目标，古田、福鼎被授予省级计生优质服务先进县称号，柘荣、霞浦、屏南、蕉城基本达到优质服务先进县标准。国土资源管理得到加强，耕地保护尤其是基本农田保护力度加大，土地整理工作扎实推进，连续6年实现耕地占补平衡。环境保护和生态建设稳步推进，基本完成市长环保目标责任制年度计划，森林覆盖率65.8%，重视环境质量建设，石板材加工污染及"青山挂白"、乱滥建寺庙教堂治理工作都取得成效，三都澳海域网箱养殖带来的污染淤积也在着手清理中。人事、编制、审计、统计、广播电视、新闻出版、外事、侨务、对台、库区、地震、水文、气象、农机、档案、地方志、老龄、残疾人事业及关心下一代工作等也都取得新成绩。

7、优化社会环境，精神文明和民主法制建设卓有成效。城乡精神文明创建活动深入开展，市本级及6个县（市、区）创建文明城市工作通过省级初评。举办了纪念闽东苏区创建70周年活动，进一步弘扬老区精神。重视少数民族地区发展，维护宗教领域安定稳定，民族、宗教工作得到加强。双拥、民兵预备役、人防、海防、国家安全等工作扎实有效。基层民主不断扩大，村居自治和村务、厂务、政务公开工作继续加强。认真做好《行政许可法》贯彻实施工作，依法治市扎实推进，"平安宁德"建设和"百千万"基层普法依法治理活动深入开展，社会治安综合治理不断加强。"严打"整治斗争、打击走私与整顿和规范市场经济秩序取得成效。全市安全生产形势趋于好转，未发生特大安全事故。突出信访问题及群体性事件得到及时妥善处理，有效维护了社会安定稳定。

8、牢记为民宗旨，政府自身建设进一步加强。围绕建设人民满意的政府，加快政府职能转变，行政审批制度改革取得阶段性成效。认真执行市人大及其常委会的决议、决定，自觉接受其法律监督、工作监督，办理人大代表建议119件。重视支持人民政协履行职能，办理政协提案172件。加强同民主党派、工商联、无党派人士和各人民团体的联系，充分发挥工会、共青团、妇联等群团组织作用。深入开展"创建效能型、服务型、责任型政府，营造优良政务环境"活动，推进绩效评估试点工作，促进政府工作提速。加大对机关作风、服务效率的督查力度，依规查处了损害投资软环境的人和事，机关工作效率和服务质量得到提高。

过去一年取得的成绩来之不易，得益于中央、省委、省政府和市委的坚强领导，离不开市人大、市政协的大力支持和有效监督，凝聚着全市人民的辛劳和智慧。在此，我谨代表宁德市人民政府，向辛勤工作在各行各业的全市人民，向给予政府工作积极支持的人大代表、政协委员、各民主党派、工商联、各人民团体、无党派人士和离退休老同志，向为宁德改革发展稳定作出贡献的中央及省属驻宁机构、驻宁部队、武警官兵、民兵预备役官兵、公安政法干警，向关心支持宁德发展的港澳同胞、台湾同胞、海外侨胞、国际友人和各界人士，表示衷心的感谢和崇高的敬意！

在肯定成绩的同时，我们也清醒地看到，我市经济生活中还存在不少困难和问题。经济总量小，支柱产业支撑力不足，科技创新能力不强，产业链短，集群效应弱，后劲项目还不多；农业产业化水平还比较低，农产品深度开发和转化增值潜力尚未充分发挥，农村基础设施相对滞后；区域经济发展不平衡，财政困难的状况未能得到根本缓解，中心城市发展相对滞后，聚集、辐射和带动作用发挥不够；社会就业压力增大，社会保障任务还很重；社会治安、安全生产形势不容乐观；一些部门工作人员行政效率不高、工作落实不力、执法行为不够规范等问题还没有根本解决，消极腐败和铺张浪费现象仍然存在，软环境建设还有待加强。对这些问题，我们都要有足够认识，深入研究，用改革和发展的办法，采取综合措施认真解决。

二、认真做好2005年各项工作，加快推进海峡西岸经济区东北翼增长极港口中心城市建设

2005年是全面实现"十五"计划目标和衔接"十一五"发展的承上启下之年，也是我市全面建设海峡西岸经济区东北翼增长极港口中心城市的重要一年。我市作为福建"延伸两翼"中的重要一翼--东北翼，又是"对接两洲"中的长江三角洲的前沿区域，应按照全省新一轮发展的基本构想、基本思路、基本态势和基本格局，充分发挥在海峡西岸经济区中的承接、传导作用，凝聚全市力量，加快东北翼增长极港口中心城市建设。这是统领我市当前及今后一个时期的奋斗目标和中心任务。全市上下应统一认识，统一行动，万众一心，开拓进取，努力实现宁德新跨越。根据中央和全省的部署精神，今年政府工作的总体要求是：以邓小平理论和"三个代表"重要思想为指导，认真贯彻党的十六大、十六届三中、四中全会精神和省委七届七次、八次全会以及市委一届十次、十一次全会精神，用科学发展观统领全局，落实宏观调控政策，紧紧抓住重要战略机遇期，推进改革开放，转变增长方式，继续实施项目带动，加快产业集聚，做大中心城市，发展县域经济，转变政府职能，改善人民生活，维护社会稳定，构建和谐社会，实现经济社会全面协调可持续发展，确保全面和超额完成"十五"计划各项目标任务。全市经济和社会发展的主要预期目标是：地区生产总值增长11%，力争更快更好一些；全社会固定资产投资增长18%；社会消费品零售总额增长11%；外贸出口增长16%；实际利用外商直接投资增长10%以上；地方财政收入增长11%；城镇居民人均可支配收入增长8%；农民人均纯收入增长5%以上；居民消费价格指数总水平控制在104%左右；城镇登记失业率控制在4.5%左右；人口自然增长率控制在7‰以内。按照上述总体要求和目标，今年政府工作必须扎实抓好以下八个方面：

1、关注农村，关心农民，提高农业综合生产能力

加快农业结构调整和农业产业化步伐。继续加大对粮食生产的扶持力度，充分调动农民务农种粮和各级政府重农抓粮的积极性，确保全年粮播面积和粮食产量继续稳步增长。按照建设农业"两带一区"和发展"六大基地"的总体要求，加大对优势农产品基地的政策和资金扶持力度，促进规模经营。狠抓名优茶生产，扩大无公害、绿色、有机茶生产规模。大力发展草生菌，推广珍稀和反季节食用菌生产，推进食用菌精深加工。调整水产、畜牧业结构，发展抗风浪大型深水网箱养殖，走生态型畜牧业发展路子，实现品种结构多样化、优质化。大力扶持蔬菜、水果、药材等种植业的产业化发展。实施林业"三、四、五"工程，调整林种结构，发展林副产品精深加工，提高林业附加值。依托优势产业和特色农产品，扶持壮大一批竞争力强、带动力大的龙头企业，重点扶持宁德南阳、福鼎海鸥、古田食用菌批发市场等一批国家和省市级龙头企业，争取全市年销售额5000万元以上的农业龙头企业增加到30家以上，年销售额亿元以上的达4家。推进农产品品牌建设，突出抓好茶叶品牌整合和统一创牌工作，提高知名度和竞争力。加强产品质量检测检验体系建设，引导、支持和帮助龙头企业开展质量、安全卫生和环保体系认证，带动和辐射全市农业标准化建设。

改善农业和农村生产条件。继续推进农村水利"六千工程"，确保完成省里下达的年度建设任务。突出抓好农村和海岛、半岛供水工程，确保完成160个，力争200个。加快农村道路硬化，确保完成700公里，力争更多些。加强防灾减灾体系建设，启动金马海堤、金港海堤和市防汛指挥中心二期工程建设，加快赛江洪水预警报系统二期工程和海堤四期强化加固工程建设，继续实施福鼎、福安、古田3个第四批初级水利化县建设和屏南、寿宁、周宁3个农村电气化县建设，提高农业防灾抗灾能力。抓好气象、水文和农机推广工作，改善农业生产条件。创新农村工作机制，继续抓好干部驻村任职工作。积极支持农民专业合作经济组织和农产品行业协会发展，完善农村社会服务联动机制，提高服务水平。加强村级农技员队伍建设，落实好津贴补助政策。同时，继续推进通讯、供电、供水、信息网络向农村延伸覆盖，不断提高农村基础设施配套水平。

千方百计增加农民收入。积极引导和支持农民面向市场开展多种经营，发展效益农业。全面开展农村劳动力转移的调查统计，启动农村"一户一工"工程。进一步建立健全各级劳动力转移工作网络，推动农村剩余劳动力转移，力争全年培训完成转移农民工5万人以上。加快集体林权制度改革，确保年内全面完成任务。认真执行促进农民增收减负的各项政策。切实解决好建设领域拖欠工程款和农民工工资问题，保障农民工合法权益。

2、突出整合提升，突显聚集效应，提高工业发展水平

进一步壮大传统优势产业。按照上下游延伸与产业配套的思路，强化分工协作，激活、带动一批中小企业发展，培育形成较为完善的产业链和相互依存、共同发展的产业群。电机电器产业要突出质量监管，以质量整合资源，以质量整合品牌，加快结构调整和做强做大，加强行业管理，依靠技术进步，强化分工协作，延伸产业链，提高产品知名度，增强竞争力。船舶修造产业要加大骨干企业船坞、船台技改力度，推进现有企业与省内外大型船舶企业的横向合作，带动中小型修船企业发展，逐步构建以造为主，修、拆为辅的产业发展格局。食品加工产业要立足资源优势，开发绿色食品，重点发展优势农产品深加工，培育建设具有地方特色的绿色食品加工基地。制药产业要重点支持柘荣、古田、福鼎药业加快扩大升级。汽摩配件工业要开发具有较高技术含量和附加值的产品，重点加快建设福鼎化油器生产基地和霞浦三沙汽摩配件生产基地。建筑建材产业要以石材加工业为重点，鼓励骨干企业提高技术装备水平，优化产品结构，促进资源合理开发利用和行业可持续发展。同时，发挥项目对生产要素的聚合作用，围绕传统优势产业，抓紧谋划和实施一批工业技改项目。重点抓好10个投资3000万元以上的工业新增长点项目，推动传统产业改造升级。

加快推进产业聚集和提升。发挥海洋和港口优势，大力培育发展临海工业，集中力量支持在建能源项目建设，争取引进一批符合国家产业政策的临海钢铁、石化等重化工业，努力打造新兴临海产业板块。按照区域集中、产业集合、要素集聚、开发集约的要求，突出抓好现有11个工业集中区，加强基础设施配套建设，推广项目"飞地"政策，促进工业项目向园区集中，使园区真正成为承接项目、资金等生产要素的主要载体。实施大企业、大集团和品牌战略，以骨干企业和名牌产品为核心，以资产为纽带，推动联合。今年重点引导和扶持安波电机、海鸥水产、三祥冶金、闽东力捷迅药业、华美汽摩配件等一批企业组建股份公司，并向集团化方向发展。

3、扩大投资，扩展消费，增强内需拉动作用

下大力气推动投资增长。继续实施项目带动，努力突破项目前期工作，加大后续项目的储备、开发和建设、实施，优化项目结构。今年计划安排重点在建项目44个，总投资300.2亿元，年度计划投资63.7亿元（温福铁路未列入），比去年增长43%；预备重点项目15个，总投资39.7亿元。健全完善领导挂钩重点项目制度，实行从前期工作到开工建设的全程负责制，加强协调调度，确保目标任务到人，项目落实到位。

进一步完善交通、能源等基础设施体系。集中力量打好交通攻坚战，加快构建"南联北接、西进东出"的现代立体交通运输网络。路网建设方面，今年重点推进温福铁路建设，沿海县（市、区）要全力做好征地、拆迁和安置补偿等工作，为铁路建设创造良好的施工环境。继续完善市内交通网建设，建成古田环湖公路、湖塘坂至柘荣公路柘荣段；完成杨太旅游公路路面硬化，确保"十·一"黄金周前全线通车；开工建设宁屏二级公路蕉城段和国道鼎柘二级公路福鼎段，确保2006年建成通车；做好沿海大通道201省道统一规划、分步实施工作，今明两年重点实施沿海疏港公路，加快建设福鼎杨岐码头至秦屿、蕉城礁头至城澳、福安湾坞互通至半屿疏港公路，确保年内竣工使用。霞浦盐田至溪南疏港公路也要加快前期工作。继续抓好宁（德）上（饶）高速公路及连接线前期工作，争取2006年开工建设。进一步深化宁（德）衢（州）铁路、京福高速公路古田连接线等一批重大交通项目前期工作，争取进入国家规划盘子。港口设施方面，认真实施港口总体规划，深化港口建设管理体制改革，统筹安排、科学利用全市港口岸线和陆域资源。继续做好城澳一类口岸配套工程建设，争取上半年通过国家验收，并积极争取申报城澳港更名为宁德港。同时，抓好城澳5000吨级滚装码头和大唐5万吨级煤码头续建，完成下白石、杨岐3000吨级滚装码头和白马门港区5万吨进港航道工程建设。电力能源方面，重点抓好宁德火电厂建设，争取年底首台机组并网发电，加快宁德核电厂前期工作。继续推进宁德洪口电站等一批水电开发项目，建成投产穆阳溪梯级二级周宁电站、古田双口渡电站、寿宁牛头山电站一期、周宁后垄溪电站一期及屏南上培电站技改工程，年内实现新增装机容量99万千瓦。同时继续抓好宁德电力输配电网络建设与改造工程，增强电力供应能力。

积极开拓城乡消费市场。不断提高服务业发展水平，努力培育新的消费热点。大力发展旅游产业，全面启动宁德、福鼎创建全国优秀旅游城市工作，重点加快太姥山、三都澳、九龙漈·鲤鱼溪和翠屏湖四个国家4A级旅游景区的申报工作，做好古廊桥申报世界文化遗产、太姥山申报世界地质公园、白水洋申报国家地质公园等各项工作。同时，围绕"一轴两带三区"和"十大精品工程"的布局，坚持开发与保护并重，整合资源，串点连线，完善六大要素，提高接待能力，加强同周边地区的旅游线路对接，推进宁福温三市及闽东北五市旅游区域合作，促进旅游消费持续升温。适应信息化潮流，积极发展信息服务业，推动企业、政府、家庭上网。做好商业网点规划，建立完善城乡商业网点网络和商品市场体系，拓宽商品流通渠道。顺应城乡居民消费需求变化的新特点，通过组织开展贸洽会、定货会、新产品展示会等各种形式的营销活动，促进住房、汽车、教育、餐饮等方面的消费继续走旺，培育体育健身、休闲养老、文化娱乐、卫生保健等方面的消费。努力开拓农村消费市场，鼓励和引导商业服务企业深入农村集镇发展连锁经营、超市、便利店等现代流通业态。全面加强市场监管，建立食品、药品安全准入机制，依法严厉打击制假售假、欺行霸市、哄抬物价等违法行为，改善消费环境，增强城乡居民消费信心。

4、做大中心城市，做强县域经济，推动区域经济协调发展

提升中心城市发展水平。根据新一轮中心城市总体规划，突出以港兴市、港城互动，加快实施中心城市"面海、向海、跨海"发展战略，推进城市"东扩面海、北展南移"。以工业为主导，以项目为抓手，在注重抓大项目的同时，全面加快蕉城区、东侨开发区和三都澳开发区建设步伐。在提升壮大食品、建材、陶瓷、化工等传统产业的同时，坚持运用"飞地"投资政策与扶持发展工业卫星镇并举，加快引进一批工业项目，重点抓好闽东工业园区和东侨开发区已入园项目的实施，争取尽快建成投产。充分利用中心城市的区位和交通优势，积极发展商贸物流、特色旅游和房地产业等城市服务业，进一步提升中心城市经济实力。按照2004-2005年中心城市建设目标，全面启动中心城市建设，把今年作为城建项目大会战年，集中人力物力财力加速83个城建项目实施，确保顺利完成年度建设任务。突出抓好以东侨开发区路网为重点的一批城市道路工程，以金港围海工程、城区防洪挡潮工程和污水处理厂、垃圾焚烧厂为重点的一批市政设施工程，以市文化中心、宁德师专新校区、市医院搬迁为重点的一批公益事业工程，以环东湖生态景观休闲区为重点的一批生态环境工程。坚持建管并举，建立科学管理城市的长效运行机制，强化以交通、卫生、市容市貌、市场秩序、小区管理等为重点的城市综合管理，提高城市管理效能。

推动中心城市和县域经济联动发展。按照"北承南联、对接两洲，西进东出、拓展腹地，区域统筹、整体推进，全面融入、服务大局"的发展布局，在着力增强宁德中心城市聚集辐射功能的同时，全面落实支持县域经济发展的政策措施，引导县（市）做大优势产业，推进工业化、城镇化进程，不断壮大经济实力。沿海县（市）要充分发挥区位、产业、港口等优势，依托便利的交通条件，率先承接发达地区产业、资金、技术转移，加速工贸经济、海洋经济和外向型经济发展，不断增强自身发展带动能力，促进沿海城市群的形成。山区五县要主动呼应融入全市发展大格局，进一步挖掘自身优势和潜力，尽快打通山海通道，发挥腹地作用，大力发展资源转化型工业、绿色农业和生态旅游业，缩小与沿海的发展差距，实现与沿海互联互动、共赢共荣，逐步构建中心城市龙头带动、县域经济多点支撑的区域协调发展格局。

5、推进改革，推动开放，不断释放发展活力

加快体制机制创新。切实抓好国有资产管理体制改革。加强国有资产监管，强化国有资产营运，促进国有资产的整合、流动、重组和存量盘活，为各级政府筹措建设资金发挥作用。继续深化闽东电力股份公司改革，抓好市国投公司、城投公司和土地收储中心的运作，加快推进内贸、外贸、建筑、农口等行业国有企业改革，改善企业内部治理结构，探索建立有效的管理体制和经营机制，确保国有资产保值增值。同时，抓好投资体制改革。全面落实国务院关于投资体制改革的决定，规范核准制，健全备案制，真正落实企业投资自主权，推进代建制和经评审的最低价中标办法试点。继续激活社会投资，推出一批项目，鼓励和引导民间资本参与建设，形成政府投资带动社会投资的良性机制。

着力推进民营经济发展。按照省委、省政府提出的"五个引导"的要求，制订出台加快民营经济发展的政策措施，在民营企业制度创新、联合重组、资本扩张和要素聚集等方面，进一步加强引导和扶持，帮助民营企业解决用电、用地、用水和资金上的需求，积极构建支持民营经济发展的服务体系。放宽市场准入，鼓励和支持民营资本进入基础设施、垄断行业、公用事业以及法律法规未禁止的其他行业和领域，努力创造一视同仁、平等竞争、共同发展的投资环境，形成有利于动员和启动民营资本投资的政策和机制，充分释放民营经济的发展潜力。

提高对外开放水平。进一步加强招商引资，继续精心组织办好宁德招商节等系列招商活动。坚持国内与国外、市内与市外并举，采取灵活务实的多种招商形式，扩大引资规模。广泛吸收社会资本、金融资本参与新区、园区、港区等载体建设，培育一批招商引资的重点区域。严格落实招商责任制，扎实抓好签约项目的跟踪落实，加强对签约项目的实际到资率和开工、竣工的考核，不断提高招商引资实效。继续做大出口规模，坚持出口市场多元化战略，进一步扩大电机电器等优势工业产品和茶叶、食用菌、水产品、果蔬等绿色农产品出口规模。培育更多的外贸经营主体，积极发展加工贸易，鼓励更多的企业拓展境外经贸业务，建立生产加工基地。抓紧做好入世过渡期结束的各项应对工作，努力提高应对国际贸易壁垒的能力，促进外贸出口持续快速增长。积极扩大宁台经贸往来，争取正式批设对金马澎地区货运直接往来口岸。

加强区域经济协作。放大区位优势，推进南联北接，加快构建南北双向融合、两面受益的区域协作发展格局。重点加强以福州市为龙头的"闽东北五市"区域经济协作和以温州市为龙头的"闽浙五市"协作联谊。着眼建立长效机制，进一步健全政府高层联席会议制度、专业部门和市县对口联系制度，从具体项目、具体工作入手，通过项目推介、对口帮扶、山海协作、旅游促销等多种途径，构建利益共同体，使区域协作富有生机和活力。

6、优化结构，优化投向，实现财税金融与地方经济的良性互动

财政工作要突出财源培植、结构调整、体制改革、财税监督四个重点。积极寻找和培育新的财源增长点，因地制宜确定市县乡三级财源建设重点。严格治税，应收尽收。严肃财经纪律，严格控制支出，集中财力保证重点支出的需要。继续深化财政管理体制改革，今年起全面实行部门综合预算，着手开展国库集中收付制度试点工作，继续扩大政府采购覆盖面。同时，加大政府债务清偿力度，建立政府债务预警机制，实行政府、部门举债审批制度和偿债责任制。

金融工作要认真贯彻稳健的货币政策。紧紧围绕扩大内需和调整结构，进一步加强银政、银企沟通合作，搞好项目对接，鼓励银行加快业务创新，拓展服务领域，加大对我市优势产业、重点项目、骨干企业和中小企业、民营经济的支持。继续深化金融改革，重点推进农村信用社改革。进一步建立健全中小企业信用担保体系和信用评价体系，重点加快组建市本级、霞浦、古田、寿宁等四家中小企业信用担保公司，提高担保基金的运作规模和质量。同时，注重改善金融生态环境，加强金融监管，维护金融稳定，防范和化解金融风险。

7、注重统筹，注意协调，全面发展各项社会事业

加快发展科教和人才事业。深化科技体制改革，加快科技创新和科学普及，继续推动"科技兴业"示范工程，引导企业进行技术革新、产品创新和工艺更新，支持有条件的企业建立研发机构和技术中心，加速科技成果向现实生产力转化。全面落实全省教育振兴行动计划实施意见，依法加大教育投入，确保预算内教育经费达到"三个增长"要求。不断巩固"两基"成果，加快推进"双高普九"。大力加强农村教育，改善办学条件，积极稳妥地进行布局调整，力争完成农村中小学危房改造年度计划。加快发展高中阶段教育，重点加强职业教育，推进职业学校并校整合，促进普通高中和中等职业教育协调发展。稳步发展高等教育，突出抓好宁德师专新校区建设，努力推进宁德学院筹建工作。深化办学体制改革，积极发展民办教育。严格规范教育收费，健全资助贫困学生的制度，确保3年内使低保家庭学生全部享受"两免一补"。深入实施人才强市战略，认真落实和完善人才政策，改善人才成长环境，逐步建立留住、吸引和用好人才的长效机制。

强化政府公共卫生管理职能。加快公共卫生体系建设，抓好宁德医院新建前期工作，确保年内开工。继续推进市县两级疾控中心和传染病区（科）建设，充实卫生应急队伍，切实加强重大传染病的预防和控制工作，不断提高公共卫生事件应急处置能力。积极稳妥推进城乡医疗卫生管理体制改革，大力发展民办医疗机构和社区卫生服务。按照加强农村卫生工作责任状的要求，认真落实有关政策，加大农村卫生投入，力争年内完成50%乡镇卫生院危房改造任务和全部卫生所"空白村"建设任务。建立和完善卫生执法监督体系，保障群众的身体健康和生命安全。

推进文化和体育等事业发展。深化文化体制改革，加快文化设施建设，重点建设宁德文化中心博物馆、图书馆等一批标志性的城市文化设施和示范性的乡镇标准文化站。加强文化市场管理，加大网吧、音像市场等日常监管力度。强化文物和民族民间文化遗产的挖掘、抢救和保护工作。扎实推进续修新志和依法修志。加强广电数字化建设。广泛开展全民健身活动，加快建设市体育中心二期工程，促进屏南、霞浦等县体育场馆建设。认真筹办第二届全市运动会，积极备战第十三届省运会。同时，认真开展全国第一次经济普查，做好"十一五"规划和2020年远景目标的编制工作。

加强人口、资源和环境保护工作。继续深入开展创建计生优质服务先进县活动，全面落实各项计生政策措施，落实农村部分计生家庭奖励扶助制度和村级计生管理员享受省级财政定补制度，突破流动人口计生管理、社会抚养费征收和"一男超生"的难题，进一步提升人口计生工作整体水平。认真做好新一轮土地利用总体规划的修编工作，严格国土资源管理，加强耕地保护。建立项目用地准入机制，推进集约、节约用地。规范各类用地行为，坚决防止乱占滥用耕地。加大闲置土地处置力度，积极实施土地整理，重点加快福鼎番岐头、霞浦福宁湾（沙头）、陇头、福安湖塘等围垦项目建设，抓紧做好铁基湾、渔井、漳湾、金港、三屿围垦的报批工作，保证项目用地需求，保持耕地占补平衡。加强港区和规划港区红线内的土地管理，确保港口和临港工业建设用地。强化海域管理，认真实施海域使用证、海洋功能区划和海域有偿使用三项制度。依法严格保护和合理开发森林、水力、矿产等自然资源。大力发展循环经济，推广节能技术、清洁能源和水循环利用。全面落实市长环保目标责任书规定的各项目标任务，加强生态环境保护，推进自然保护区和柘荣、霞浦、周宁国家级生态示范县建设。继续推进殡葬改革，加大力度开展"青山挂白"专项治理，加强对重点流域、重点区域的生态治理，重点整治三都澳海域网箱养殖、石材业污染等突出问题。做好湿地、生物多样性保护和水土保持工作，加强沿海防护林体系建设和生态公益林保护，推进城乡绿化一体化，打造"绿色宁德"。

8、维护群众利益，维护社会稳定，努力构建和谐社会

切实关注民生，不断改善人民生活。做好就业和再就业工作，继续实施"16833"就业工程，加强培训和技能鉴定，提高劳动者的就业能力、创业能力和工作转换能力。确保全年新增就业岗位2.6万个，帮助6000名下岗失业人员实现再就业。进一步建立健全以养老、失业、医疗保险为主要内容的社会保障制度，使全市城镇职工基本养老保险、失业保险、医疗保险参保率分别达到90%、85%和80%。继续抓好农村低保工作，完善城镇居民最低生活保障制度，全面落实农村五保供养政策。改进和完善征地补偿办法，逐步建立失地农民的基本生活保障制度，保证被征地农民原有生活水平不降低。努力改善离退休老干部生活条件。继续发展社会福利和慈善事业，加强社会救助，发展老龄事业和残疾人事业。加大扶贫开发力度，重点抓好省市扶贫开发重点村和8个畲族乡的挂钩帮扶工作，落实扶贫项目、资金和产业扶贫信贷政策，帮助贫困人口尽快脱贫致富。启动老区工作优先行动，认真落实进一步加快老区建设的若干意见，加快畲族经济开发区发展步伐，重视加强库区工作，在资金、项目上倾斜扶持老区、库区和少数民族地区发展。继续实施"造福工程"和滑坡险情村搬迁，确保全年搬迁6000人，力争更多些。建立健全地质灾害预防体系，逐步对地质灾害点群众实施搬迁。

加强精神文明和民主法制建设，促进三个文明共同进步。广泛深入开展各个层面的群众性精神文明创建活动，不断提高城乡文明程度，努力争创省级文明城市，并争取早日跨入全国文明城市创建工作先进城市行列。深入开展公民道德教育和实践活动，高度重视加强和改进未成年人思想道德建设，积极为未成年人健康成长创造良好的环境。深化国防教育，增强国防观念，重视国防设施建设，做好民兵预备役和人防工作。深入开展双拥共建活动，进一步巩固军政军民团结。贯彻落实市委关于进一步加强人大、政协工作的决定。自觉接受人大及其常委会的法律监督和政协的民主监督。重视和发挥各民主党派及工商联的作用。认真做好人大代表议案、建议、意见和政协委员提案的办理工作，加强督查落实，提高办理质量。支持工会、共青团、妇联等群团组织发挥作用。扎实做好新形势下的民族、宗教、侨务和对台工作。继续深化基层"百千万"普法依法治理活动，进一步提高公民法律素质，推进依法治市。加强基层民主政治建设，深化政务公开、村务公开，切实保障广大人民群众依法行使民主权利。

扎实推进"平安宁德"建设，全力维护社会安定稳定。进一步加强社会治安综合治理，实施社区（村居）"11221"工程，设法解决县、乡两级综治机构警力不足和经费保障问题，努力构建治安防控体系。严密防范和严厉打击各种邪教组织、地下宗教势力和各种敌对势力的渗透破坏活动。保持对严重刑事犯罪活动的严打高压态势，继续开展对"两抢两盗"、"黄赌毒"、"六合彩"等突出问题的专项治理。严格落实安全生产责任制，建立安全事故预警机制，加强安全生产应急救援体系建设，努力消除各类重大安全隐患，坚决遏制重特大安全事故的发生。高度重视群众信访，加强矛盾纠纷排查调处工作，积极预防和妥善处置群体性、突发性事件，努力创造更加稳定和谐的社会环境。

三、推进对人民负责的政府建设，不断提高政府行政能力

全面完成今年各项任务，确保"十五"计划目标的顺利实现，任务繁重，责任如山。各级政府要以开展保持共产党员先进性教育活动为契机，进一步强化责任意识，认真履行职责，推进对人民负责的政府建设，努力提高政府行政能力和水平，做到对党负责、对事业负责、对人民负责，切实担负起人民赋予的重任。

提高推动发展的能力。发展是政府的第一责任。各级政府要把提高发展经济的能力作为执政能力建设的第一目标，善于把握市场经济的内在要求和运行特点，遵循规律，顺势而进；善于把握重要战略机遇期，用好机遇，抢占先机，赢得发展；善于把握经济形势的发展变化，未雨绸缪，正确应对，创新办法，趋利避害，突破风险，破解难题，以经济社会发展的新成果来体现执政能力建设的新成效。

提高科学决策的能力。要吃透"上情"，准确把握上级精神，确保中央、省、市的各项决策部署落到实处，确保政令畅通。要摸清"下情"，准确把握基层意愿，尤其是对事关全市经济社会发展的重大事项，要倾听各方面的意见和建议，广泛开展调查、研究、论证，避免决策的盲目性和随意性。要掌握"社情"，建立健全重大问题集体决策制度、专家咨询制度、社会公示和听证制度，提高决策透明度和公众参与度。

提高务实为民的能力。要改进作风，把狠抓落实、为民谋利作为工作的关键点，凡是得民心、顺民意的事情，都要竭尽全力去做，凡是失民心、悖民意的问题，都要雷厉风行去改，切实把各项工作落到实处，抓出成效，干出水平。特别是对就业、社保、农民工工资等群众普遍关心的难题，要勇于解决，善于解决，及时解决。要创新抓落实的办法，实行严格的目标责任管理，完善交办、催办、督办、查办等制度。要强化部门协调联动，条块之间、行业之间要顾大局，讲默契，重合力，同心协力抓落实。

提高依法行政的能力。坚持依法办事，严格按照法定权限和程序履行职责，是对政府各部门和全体公务员的基本要求。要牢固树立法治观念，认真落实《行政许可法》和《全面推进依法行政实施纲要》，不断提高依法行政、依法办事的自觉性。进一步规范行政行为，在行政方式上坚持法治原则，在宏观决策上考虑法律效果，在具体管理中牢记依法办事，把依法行政贯穿于政府行为的全过程，促进政府行政行为的法制化、规范化。强化行政执法监督，建立实行执法过错责任追究制，努力做到严格执法、公正执法、文明执法。

提高营造环境的能力。突出政府职能转变，继续深化行政审批制度改革。注重运用经济和法律的手段，改进经济调节和市场监管方式，努力创造良好的发展环境，更好地为市场主体服务。重视履行社会管理和公共服务职能，把精力更多地放在推进社会发展和解决民生问题上，集中更多的物力和财力解决人民群众关心、改革发展急需、社会稳定关键的问题。进一步优化政务环境，创新政府管理方式和服务方式。加强市政府网站建设，推进电子政务，缩短办事程序和时间，降低行政成本，提高行政效率，建立政府提速长效机制。全面推进绩效评估工作，充分发挥政府督查部门、效能监察机构的作用，严格落实责任追究制，严肃查处损害投资软环境的人和事。依法保护企业合法权益，维护好企业正常的生产生活秩序。广泛开展诚信教育，推进诚信政府、诚信社会、诚信宁德建设，着力营造高效、安全、诚信的投资创业环境。

提高从严治政的能力。坚持标本兼治、综合治理、惩防并举、注重预防，建立健全教育、制度、监督并重的惩治和预防腐败体系。各级领导干部要严格要求，以身作则，认真落实党风廉政建设责任制。全体公务人员都要经得起考验，做到自重、自省、自警、自励，始终坚持公道正派、清正廉洁，决不允许以权谋私，贪赃枉法。要坚决纠正行业不正之风，严肃查处违纪违法案件，真正树立起对人民负责、清正廉洁的政府形象。

各位代表，新的一年是充满希望的一年。开创宁德现代化建设的新局面，是时代赋予我们的神圣使命。我们对宁德的未来充满信心。我们要全面贯彻落实"三个代表"重要思想和党的十六大、十六届三中、四中全会精神，以科学的发展观为指导，在市委的领导下，紧紧依靠全市人民，团结奋进，开拓创新，为全面推进宁德海峡西岸经济区东北翼增长极港口中心城市建设而努力奋斗！
